# Supplementary material for: Longitudinal changes in structural lung abnormalities using MDCT in chronic obstructive pulmonary disease with asthma-like features
Source: PLoS One. 2019 Dec 30;14(12):e0227141. doi: 10.1371/journal.pone.0227141 (PMC6936827; doi:10.1371/journal.pone.0227141)
Supplement: Attachment — Submitted filename: Response submission(1205).docx [file pone.0227141.s003.docx]

**RESPONSE TO EDITOR:**

**EVALUATION:**

Your manuscript is acceptable for publication after small revisions that should be done on Figures. On Figure 1 please change the words "abnormal chest shadows" into something more appropriate, like "abnormal lung parenchymal lesions other than emphysematous change". On Figure 2, and also the markings for the median and interquartile range for each time point and group.

**RESPONSE:**

We greatly appreciate the editor’s constructive comments which have helped us to considerably improve the quality of our manuscript. We have revised our manuscript according to the editor’s comments.

1) We revised Figure 1 according to the editor’s comment.

Fig 1. Flow chart of the study participants

2) We also revised Figure 2 according to the editor’s comment. We have added bars at which represent the median and interquartile range (Page 11).

Fig 2. Changes in radiological parameters over two years of follow-up

Before:

After:

Page 11, lines 220-223, in Results

Before:

“Notes: Radiological parameters were compared between enrollment and two-year follow-up with the Wilcoxon signed-rank test. WA% shows the mean wall area percentage at the 5th generation of bronchi.”

After:

“Notes: Radiological parameters were compared between enrollment and two-year follow-up with the Wilcoxon signed-rank test. WA% shows the mean wall area percentage at the 5th generation of bronchi. The thick horizontal bars at the sides of each graph show the median, while the thin horizontal bars at the sides of each graph show the interquartile range.”
